# Response to comment on “Using genomic data and machine learning to predict antibiotic resistance: A tutorial paper”

**DOI:** 10.1371/journal.pcbi.1014710

**Published:** 2026-09-18

**Authors:** Lucy Moctezuma Tan, Faye Orcales, Pleuni Pennings

**Affiliations:** 1 Department of Statistics, California State University East Bay, Hayward, California, United States of America; 2 Biological and Medical Informatics Graduate Program, University of California San Francisco, San Francisco, California, United States of America; 3 Ecology and Evolution of Communities Team, ISEM, Univ Montpellier, CNRS, IRD, Montpellier, France; 4 Department of Biology, San Francisco State University, San Francisco, California, United States of America; SIB: Swiss Institute of Bioinformatics, SWITZERLAND

We appreciate the comment by Chicco and Jurman [1] about our recent paper [2]. The goal of our paper was to make it easier for students and researchers to get started with machine learning as applied to genomic data to predict drug resistance.

In our paper, there is a section on how to evaluate a machine learning model where we discuss the metrics most commonly encountered in the literature and in other tutorials: accuracy, precision and recall.

When teaching model evaluation, accuracy is the most common metric to start with [3] but it should be made clear (as we, in fact, did in our paper) that accuracy can be misleading if the dataset is imbalanced. In such an imbalanced dataset where the vast majority of samples belong to one label, the model could just predict that label for all samples and get it right most of the time, leading to high accuracy while never correctly predicting the minority label. Nevertheless, in our experience, accuracy is a good starting point for a learner because after making predictions for all of the cases in the test dataset, a logical next step is to tally the number of times that the model got it right. For these reasons accuracy is a great teaching tool, but usually not the right choice for reporting in a research paper. We thus advocate for the usage of other metrics as well.

In our paper, we discussed precision and recall as important evaluation metrics. Precision is the ratio of true positives to all predicted positives and recall is the ratio of true positives to all actual positives. There are many benefits of using precision and recall, including their interpretability. Other options include the F1 score, F1-macro and the Matthews Correlation Coefficient (MCC).

However, instead of prescribing a “single best metric”, our preference is to have the students think about what they want a model to achieve. “Imagine you are a doctor” is what we often say, or “Imagine you run a ward in a hospital”. When resistant strains are rare, but failure to detect such a strain can be deadly, the most important task of a model is to identify the resistant samples correctly, whereas identifying the susceptible samples is much less crucial. In this scenario, recall for resistance is the most important metric.

Here we will give a brief overview of the pros and cons of the following metrics: Accuracy, Recall, Precision, F1 scores, the F1-macro score and MCC, which were touched on in the original article and the subsequent comment.

We use **accuracy** for a dataset with balanced target labels, for example, a similar number of resistant and susceptible isolates to any specific antibiotic drug. As mentioned before, leaning on accuracy only can be deceiving when the dataset is not balanced. Accuracy also gives the same weight to different kinds of errors, false positives and false negatives, whereas the person using the model may weight false positives and false negatives differently.

When a model user is particularly interested in preventing false positives or false negatives, they should use **precision** or **recall** respectively. For example, if our model is used to classify emails into spam (positive class) and legitimate emails (negative class), then we want to prioritize precision because it minimizes false positives (a false positive here means that a legitimate email ends up in the spam folder). On the other hand if we use a machine learning model to detect a serious disease, we want to make sure we err on the safe side and not miss any potential cases (those missed cases are false negatives) so we would prioritize recall for disease.

If a user is interested in balancing the performance of both metrics, precision and recall, **F1** is the obvious choice. The F1 score is the harmonic mean between precision and recall [4,5]. We use it in cases where we want to avoid false positives and false negatives equally. Unfortunately, F1, just like precision and recall, is sensitive to a problem called “class swapping”, which means that the metric varies depending on which class is considered the positive class in a binary classification. To avoid the problem of class-swapping, one can calculate the F1 for the resistant class and the F1 for the susceptible class and average the two values to get the **F1-macro** score. Because F1-macro is a useful single metric that tells us how well a model performs, we used it for cross-validation in our paper [2].

Finally, the **MCC** has many characteristics in common with the F1-macro score. It considers all 4 values in the confusion matrix and class swapping does not affect its value. However, it may not be easy for non-technical users to interpret a metric that ranges from -1 to 1. Neither F1-macro or MCC scores are perfect for capturing model performances and like all metrics they have their particular drawbacks, such as being sensitive to prevalence changes, extreme imbalance, etc [3]. In Table 1, we summarize pros and cons of the metrics we discussed.

**Table 1:** Guide to selecting ML evaluation metrics in AMR prediction.

Metric	Preferred when	Avoid when	AMR example
Accuracy	- Classes are balanced	- Avoid when classes are imbalanced	You have (or create) a balanced data set of resistant and susceptible samples
Precision	- Avoiding false positives is the priority	- Avoid when missing true resistant cases is the bigger risk	You want to be absolutely sure that a strain is resistant to this drug before moving on to another drug (more expensive or more side effects)
Recall	- It is most important to not miss any resistant cases	- Avoid when false positives carry a high cost	Missing a resistant strain could lead to a hospital-wide outbreak. Better to over-flag susceptible cases as resistant than to miss a “superbug”
F1 macro (Average for F1 for the resistant class and F1 for the susceptible class)	- Classes are imbalanced - You want a score that ranges from 0 to 1 (easier to interpret with other metrics that use the same range) - You want to be able to decompose the metric (e.g., F1 score for each class) for analysis	- Avoid if you consider certain types of error to be more important	You are developing a general diagnostic tool that can work independent of which class is the majority
MCC	- Classes are imbalanced - You want a score that can capture when a model is performing better or worse than random	- Avoid when you want to decompose which class the model is having issue predicting, particularly for multiclass classification	You are developing a general diagnostic tool that can work independent of which class is the majority

The authors of the comment argue that the F1 score can be overly optimistic about model performance [1]. This can be true in some cases when only the F1 for one class is used. In most cases using F1-macro leads to more accurate model evaluation. The confusion matrix in Fig 1 of the comment (reproduced here as Fig 1) is a great way to illustrate how F1-macro can be used to evaluate a model when the data set is unbalanced.

**Fig 1:**
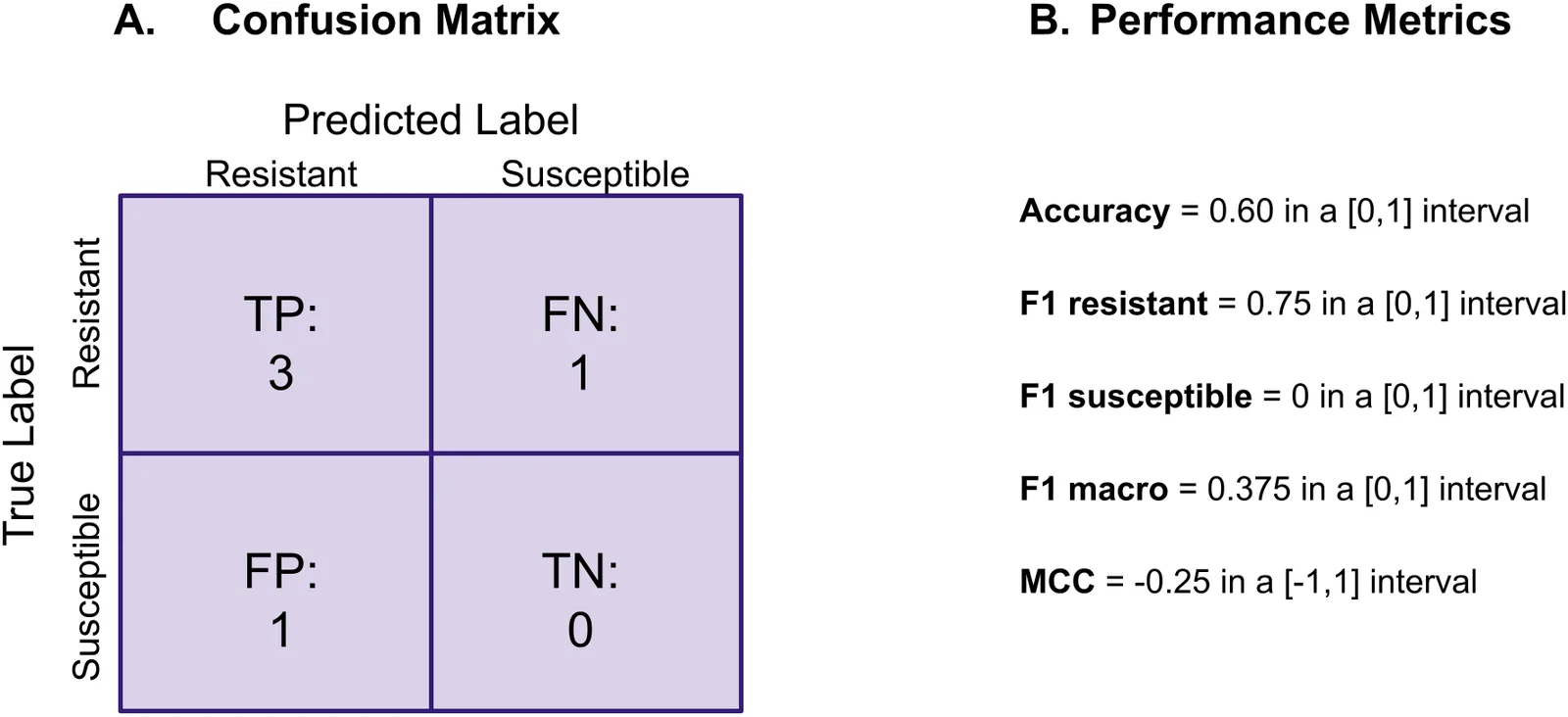
Confusion matrix with calculated performance metrics.

The example from the comment is a case of an unbalanced dataset with only one negative sample. The confusion matrix shows 3 true positives, 1 false negative, 1 false positive, and 0 true negatives. The confusion matrix shows that the underlying model doesn’t perform well because it labels the only negative sample as a positive. Despite this, accuracy and F1 for the resistant class are fairly high, which the commenters pointed out is misleading. However when we compute the F1 score for the susceptible class, we see that it is low, as it should. When there is a class imbalance the minority class usually gets a low F1 score [6] which offsets the high score from the majority class. MCC scores should be compared to F1-macro rather than individual F1 scores. As we can see, both the MCC score of -0.25 and the F1 macro score of 0.375 shows the poor performance of the model. In addition, the negative MCC score shows that the model performs worse than random guessing.

## Cross validation

In our original paper we also dedicated a section to cross-validation [2]. We decided to teach **k-fold cross validation** in our paper because it is commonly used for hyperparameter tuning. In addition we used **stratified blocked cross-validation** because of its use specifically in biology. Chicco and Jurman would have preferred we discuss **repeated hold out validation**.

To perform **k-fold cross validation**, the training dataset is split into k partitions or folds. Oftentimes, k is chosen to be 10, but in our paper we used 4 folds, which reduced compute time. In this case, one of the 4 folds is held out and used as the validation data set while the rest are all used for training. This is repeated until each unique fold has been used as the validation data set once.

The commenters pointed out that k-fold cross validation partitions the training data into folds only once and they prefer using **repeated hold out validation** or **monte carlo cross validation** instead. In this method a random subsample of the training data is held out as the validation dataset, which can be repeated many times (e.g., 1000 times).

The benefit of k-fold cross validation is that each sample is used exactly once in a validation dataset, which means that all training data is used while needing modest computing resources. On the other hand, a benefit of repeated hold out validation is that one can choose the size of the hold out and the number of repeats independently whereas in k-fold cross-validation, the choice of k determines both. Repeated hold out validation can test many more random partitions than k-fold cross-validation, which is good if one wants to estimate confidence intervals. However, repeated k-fold cross validation can also be used to compute confidence intervals. These repeated methods typically require significantly more computing resources than the standard k-fold cross-validation.

One of the reasons we presented k-fold cross-validation to students first was so that it would be easier to introduce the **stratified blocked cross-validation** technique, which takes into account the phylogenetic relationships of the isolates. It is important for researchers in the biological sciences to consider whether each isolate is truly independent [7]. For example, when studying *E. coli* bacteria, we have to consider the sequence types represented in our dataset. The dataset we used contained many isolates that belonged to sequence type 131 and it is known that many 131 strains are resistant to several antibiotics [8]. The risk here is that the machine learning model may simply “learn” that sequence type 131 is resistant, which is a type of data leakage.

When we block by sequence type, however, it forces the model to learn how to recognize resistance without relying on sequence type information. This may reduce model performance, but is done to ensure generalizability to other datasets that may have a different sequence type distribution [9,10].

Note that in our manuscript we blocked by sequence type to make the folds, but not for the initial train-test split [2]. It is also possible to do both to avoid any leakage of sequence type information, which would allow us to test the generalizability of the model on unseen sequence types. However, we note that there may also be situations where it is not a problem to let the model use sequence type information, for example if distributions of sequence types and resistance phenotypes are stable across time and space.

## Conclusion

When using machine learning to predict resistance phenotypes or to perform other tasks in biology, there are many decisions to be made – for example on how to organize the genomic data (e.g., genes, SNPs, kmers), what models to use (e.g., tree-based, logistic regression, deep learning), how to perform parameter tuning and validation and finally how to evaluate the resulting models. The latter two decisions were the topic of this exchange. While we do not provide hard and fast rules for the learner on what decision to make, we hope that this note helps clarify the options so that a learner can make an informed decision or try out several options.

While discussions on evaluation and training methods are of scientific interest and they are needed to move our field forward, the real test for any of the machine learning models will happen when their outcomes are used either for other research or indeed in the clinic. It is not yet clear whether these models can help clinicians make better treatment decisions for real life patients.

One obstacle we see for implementation of machine learning in the clinic for drug resistance predictions is that the bioinformatics pipelines are not standardized, which means that models cannot easily be trained on one dataset and then used to predict resistance for new samples [11–13].

We thank the authors of the comment for their interest in our work and we hope that readers will benefit from their and our work.
